# Disparities in suicide mortality trends between United States of America and 25 European countries: retrospective analysis of WHO mortality database

**DOI:** 10.1038/srep20256

**Published:** 2016-02-17

**Authors:** Guillaume Fond, Pierre-Michel Llorca, Mohamed Boucekine, Xavier Zendjidjian, Lore Brunel, Christophe Lancon, Pascal Auquier, Laurent Boyer

**Affiliations:** 1 Université Paris Est-Créteil Val-de-Marne, France; 2 Pôle de psychiatrie des hôpitaux universitaires H Mondor, DHU Pe-Psy, Créteil, France; 3 INSERM U955, Eq Psychiatrie Translationnelle, Créteil, France; 4 Fondation FondaMental Fondation de coopération scientifique en santé mentale, Créteil, France; 5 Department of Psychiatry, La Conception University Hospital, Marseille, France; 6 Aix-Marseille University, EA 3279 – Public Health, Chronic Diseases and Quality of Life - Research Unit, 13005 Marseille, France; 7 CHU Clermont Ferrand, Clermont Ferrand, France

## Abstract

The objective was to examine changes in temporal trends in suicide mortality in 26 Western countries by retrospective trend analysis of the WHO mortality database on causes of deaths. From 1990 to 2010, there was a median reduction in suicide mortality of 22.7%, ranging from a 46% reduction in Estonia to a 26.2% increase in Romania. Suicide mortality decreased by ≥20% in 15 countries, and the reduction tended to be greater in countries with higher mortality in 1990. In most of the central European countries mortality strongly declined. The median changes in the age groups were −25.3% (range −62.9% to 72.6%) in people aged 15–24 years, −36.9% (−60.5% to 32.4%) in 25–34 years, −3.6% (−57.1% to 92%) in 35–54 years, −12.2% (−37% to 65,7%) in 55–74 years and −16.1% (−54.5% to 166.7%) in ≥75 years. Suicide prevention programs in youths and in the elderly seem to be effective (at least in females for the elderly) and efforts should be pursued in this way. However, suicide mortality of the people aged 35–54 years has increased in half of the studied countries between 1990 and 2010. Public policies should further orientate their efforts toward this population.

The World Health Organization (WHO) published a report in 2014 stipulating that a person die every 40 seconds from suicide somewhere in the world^1^. In the year 2020, approximately 1.53 million people will die from suicide based on current trends and according to WHO estimates. Ten to 20 times more people will attempt suicide worldwide^2^. This represents on average one death every 20 seconds and one attempt every 1–2 seconds. Suicide prevention is therefore a major issue for public health policies^3^,^4^,^5^. Twenty-eight countries today are known to have national suicide prevention strategies, while World Suicide Prevention Day, organized by the International Association for Suicide Prevention, is observed worldwide on 10 September each year^1^. To be effective, these policies need to rely on exhaustive and accurate deaths rates, in order to target specific populations and assess their effectiveness as well as the impact of social context^6^.

To help countries monitoring their death rates and carrying effective public health programs, the WHO created an international mortality database, with all-causes mortality data from 1979 to 2010. The WHO mortality database is a compilation of mortality data by age, sex and cause of death, freely available at http://apps.who.int/healthinfo/statistics/mortality/whodpms/. The data is collected and submitted within 18 months following the census. The data is then checked and treated for an average of two years before publication on the website. This data is prospectively recorded for every country, thus virtually covering the entire population. Published data was only taken from medical certificates.

In this study we analyzed suicide mortality in 25 European countries and United States of America (USA) from 1990 to 2010 and examined temporal trends in suicide rates for both sexes, males and females and for subjects aged [15–24], [25–34], [35–54], [55–74] and 75+ to help decipher the effect of the contributing factors.

## Methods

Suicide deaths registered in the World Health Organization mortality database at June 2015 were extracted for European countries and USA^7^. The quality of mortality data has been evaluated by the WHO^8^. Data quality is checked annually according to the Health Facility Data Quality Report Card (DQRC). The DQRC examines completeness of reporting; internal consistency of reported data; external consistency of population data and external consistency of coverage rates (for more details see http://www.who.int/healthinfo/DQRC_Indicators.pdf). A return to the data collectors on the quality of their entered data is sent each year. Albania, Luxembourg, Monaco, Iceland and Malta were excluded for missing data. For Slovakia data was only available since 1992, for Denmark since 1994, for Switzerland since 1995, for Serbia since 1998, for Cyprus since 1999. There were not included in our analyses. For almost all other countries, data up to 2010 were available.

We used Group-based trajectory modeling^9^,^10^ to identify distinctive group of trajectories based on the assumption that the population is composed of mixture of distinct groups defined by their developmental trajectories, while recognizing uncertainty in group membership. Using SAS proc traj^9^,^10^, we model pattern of change over time in the suicide mortality rate of 26 countries (dependent variable) between 1990 and 2010. Comparisons were made between models allowing for varying numbers of trajectory groups, as well as models including time as linear, quadratic, and cubic, using the Bayesian (BIC) information criteria. More precisely, we use the BIC log Bayes factor approximation 2 log(Bij) ≈ 2*(BIC_i_ − BIC_j_) – the BIC of the more complex (large number of group, or higher order equation) less the BIC of the null (simpler) model – to select the model that better fits the data. According to Roeder and Nagin^9^ classification, a value between 0 and 2 refers to “Not worth mentioning” evidence against simple model, between 2 and 6 refers to “positive evidence against the simple model”, between 6 and 10 refers to “strong evidence against the simple model”, and higher than 10 refers to “very strong evidence against the simple model”.

Mortality values were adjusted by the age distribution of the European standard population to obtain European Adjusted Standardized Death Rates (ASDRs). After logarithmic transformation of rates, we fitted a linear regression from 1990 until 2010. We performed these analyses for both sexes and respectively for females and males, and for subjects aged [15–24], [25–34], [35–54], [55–74] and 75+.

## Results

### Overall changes in suicide mortality

**Figure 1:**
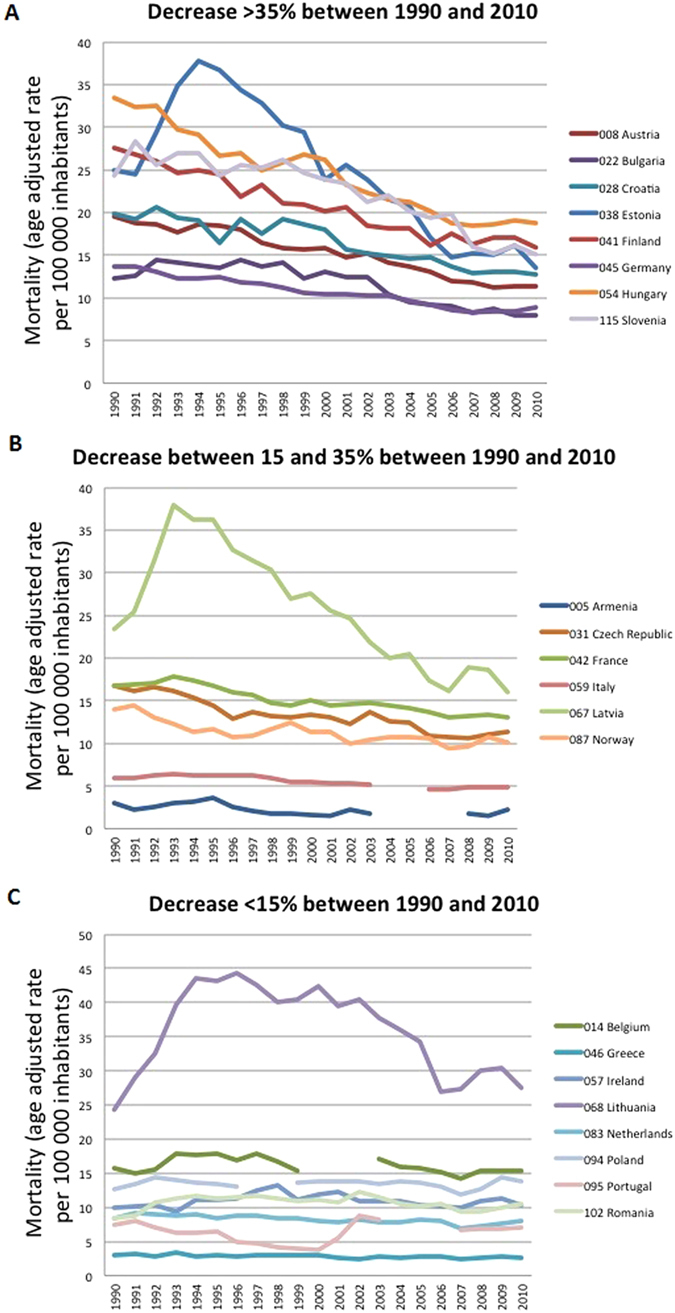
(**A**) Decrease in age-standardized death rates by suicide >35% between 1990 and 2010. (**B**). Decrease in age-standardized death rates by suicide between 15 and 35% between 1990 and 2010 (**C**). Decrease in age-standardized death rates by suicide <15% between 1990 and 2010.

Figure 1A–C and Table 1 show the changes in suicide mortality in 25 European countries and USA from 1990 to 2010. Over the 20 years, suicide mortality declined by a median of 22.7%, with individual values ranging from a 46% reduction in Estonia to a 26.2% increase in Romania. The decrease was >20% in 15 countries, and in 4 countries suicide mortality continued to increase.

**Figure 2:**
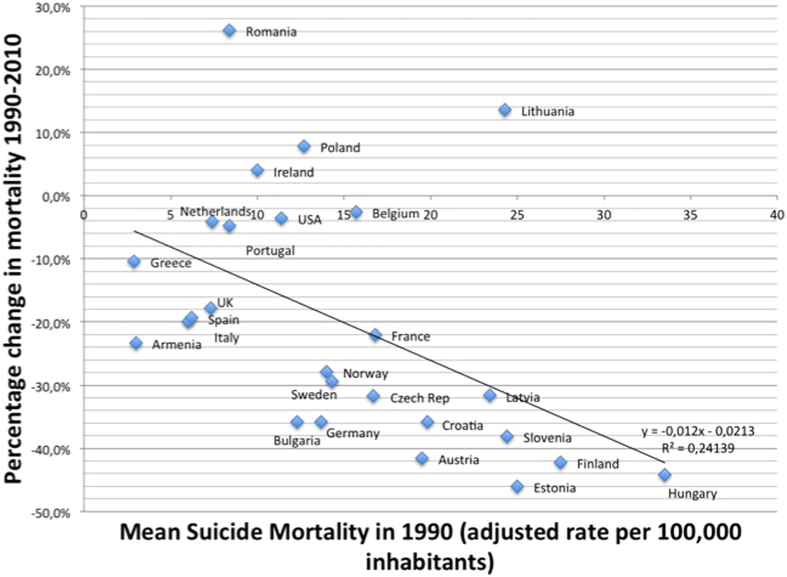
Percentage changes in suicide mortality in European countries and USA during 1990–2010 according to the mean suicide mortality in 1990.

When the 20-year mortality changes were plotted against the mean suicide mortality in 1990 (Fig. 2), countries with high suicide mortality in 1990 tended to have greater reductions in mortality. However, the decrease varied substantially. Latvia, Slovenia, Estonia and Lithuania had similar suicide mortality rates in 1990. Lithuania has a 13.6% decrease while the three other countries had a 30–45% decrease. Only a slight reduction of suicide mortality was observed in USA, Belgium, Netherlands and Portugal between 1990 and 2010 (between −2.5% and −4.8%). During this period, suicide mortality increased in 4 countries, Romania (+26.2%), Lithuania (+13.6%), Poland (+7.9%) and Ireland (+4%). There was substantial reductions >20% in Estonia, Hungary, Finland, Austria, Slovenia, Croatia, Bulgaria, Germany, Czech Republic, Latvia, Sweden, Norway, Armenia, France and Italy.

**Figure 3:**
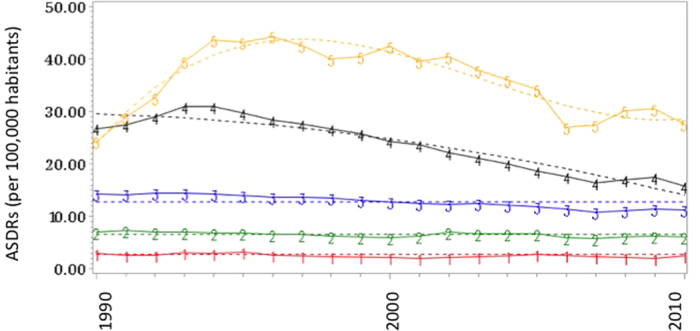
Time series of suicide attempts between 1990 and 2010 in 26 countries (25 European countries + USA) grouped by trajectories. ASDRs Age-standardized death rates. Group 1: Armenia and Greece. Group 2: Italy, Spain, United Kingdom, Netherlands, Portugal. Group 3: Austria, Bulgaria, Croatia, Czech Republic, Germany, France, Norway, Sweden, Belgium, Ireland, Poland, Romania, USA. Group 4: Estonia, Finland, Hungary, Latvia, Slovenia. Group 5: Lithuania.

With time, suicide mortality values for different countries seemed to converge. In 1990 mortality (per 100 000) ranged from 2.9 in Greece to 33.5 in Hungary (difference 31.4), whereas in 2010 mortality ranged from 2.3 in Armenia to 27.6 in Lithuania (difference 25.3). Time series were examined in Fig. 3. Five groups were identified in regard of ASDRs evolution between 1990 and 2010. The two first groups (including Armenia and Greece, Italy, Spain, United Kingdom, Netherlands and Portugal) had relatively stable suicide rates, below 10 per 100,000 habitants. The third group (including Austria, Bulgaria, Croatia, Czech Republic, Germany, France, Norway, Sweden, Belgium, Ireland, Poland, Romania and USA) had ASDRs between 10 and 15 per 100,000 habitants, with a mean slight decrease between 1990 and 2010. The two last groups (including Estonia, Finland, Hungary, Latvia, Slovenia and Lithuania) had high rates of ASDRs in 1990 (around 30 per 100,000 or more) with a strong decrease between 1990 and 2010. Except for Lithuania, the other countries ASDRs have almost joined the suicide rates of those of group 3 in 2010.

### Changes in suicide mortality by gender

Reductions in mortality were most marked in males (median change −37% (range −76% to −14%)), and reductions of 15% or more of suicide mortality in males were observed in 22 countries on 26. Reductions in female suicide mortality were more moderate (median range −20% (range −46.1% to +43%)). Increase of suicide mortality in females was observed in all countries where global suicide mortality increased (Romania (+43%), Lithuania (14.8%), Poland (+13.8%), Ireland (+12%)), and in the Netherlands (+3.6%)). In the USA, suicide mortality increased in males (+2.2%) while it decreased in females (−7.8%).

### Changes in suicide mortality by age group

**Figure 4:**
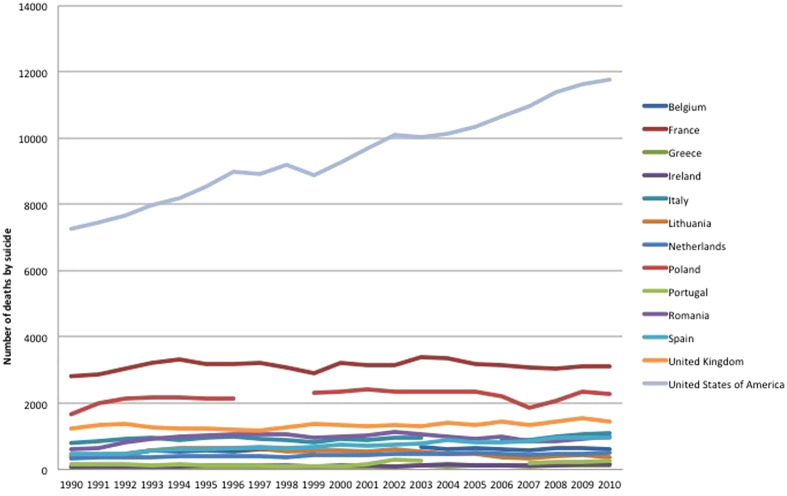
Evolution of suicide mortality in the 35–54 years males.

Reductions in mortality were most marked in people aged 25–34 years (median change −36.7% (range −60.5% to 32.4%). Reductions in 15–24 years were similar to all-ages (median change −25.3% (range −62.9 to 72.6%)). The median change of people aged 75+ was −16.1% (−54.5% to 166.7%) with suicide mortality continuing to increase in 8 countries. The slightest reduction in suicide mortality was found in the 35–54 years old, with a median of −3.6% (57.1% to 92%) and an increase in the half of countries (13/26 countries). In the USA, suicide mortality increased in the 35–54 years (+62.9%) (Fig. 4) and in the 55–74 years (+41.5%), while it decreased in the other age categories. More specifically, the suicide mortality in the 35–54 years old males increased regularly in the USA between 1990 and 2010 (Fig. 3).

## Discussion

This study shows substantial differences between European countries in temporal trends of suicide mortality, as well as differences in mortality changes with age. USA were classified 21/26 in regard of the decrease of suicide mortality between 1990 and 2010 with a decrease of 3.5%, while the median for European countries was −23.3% (range from −46% to 26.2%). Altogether, the 35–54 years old people benefited the less from suicide mortality decrease between 1990 and 2010. The sustained decline observed in many countries after 1994 seems to indicate that suicide mortality will continue to decrease beyond 2010.

### Interpretation of results

Overall, the suicide mortality has decreased between 1990 and 2010 in USA and Western European countries. Many suicide prevention programs were developed during this period^11^,^12^,^13^,^14^,^15^,^16^,^17^,^18^,^19^. Suicide mortality also strongly decreased between 1990 and 2010 in most of the Eastern European countries. These epidemiological patterns may be linked to rapid changes in suicide attempts and medical care that took place in these countries after the collapse of the communist regimes in the 1990 s^20^,^21^. This phenomenon may also at least in part explain the strong decrease of suicide mortality in Germany (−35.8%) that followed the German reunification of 1989.

Our analyses showed that mortality levels in 1990 could have influenced some of the relative differences in mortality reductions, but they cannot account for a large part of variations between countries (Fig. 2). Among medical factors influencing suicide rates, education of physicians was found as one of the most effective in suicide prevention^22^. Psychiatric disorders are present in at least 90% of suicides and more than 80% are untreated at time of death^23^. Depression is untreated or undertreated in general, even after suicide attempt^24^,^25^. Thus, treating mood and other psychiatric disorders may be suggested as a central component of suicide prevention^26^,^27^. The newer antidepressants, selective serotonin reuptake inhibitors (SSRIs) and new generation non-SSRIs introduced in the late 80 s were found to have fewer discontinuation rate^28^ and were associated with lower lethality in cases of intentional overdose^29^. Higher prescription rates of antidepressants correlate with decreasing suicide rates in adults or youth^30^,^31^,^32^. However the association between the administration of antidepressants and suicide risk remains debated to date^33^. The risk of an ecological fallacy, that is, inferring causality from group correlations, prevents attributing decreases in suicide rates solely to antidepressant use. However suicide is not only associated with depression, as all mental disorders have an increased risk of suicide excepting mental retardation and dementia^26^. Lithium showed efficacy in suicide prevention for bipolar disorders^34^ and clozapine for suicide prevention in schizophrenia^35^.

Beyond pharmacological treatments, training physicians includes also the evaluation of the risk, the emergency and the dangerousness of the suicidal episode. Restricting access to lethal means was found as a powerful tool to prevent suicide^22^. Promising results in reducing repetition of suicidal behavior and improving treatment adherence exist for cognitive therapy^36^,^37^,^38^ problem-solving therapy^39^, intensive care plus outreach^39^, and interpersonal psychotherapy^40^, acceptance and commitment therapy^41^,^42^ compared with standard aftercare.

Our results suggested that suicide rates were strongly influenced by age. Suicide is the second leading cause of death among persons aged 10–24 years in Western countries^43^. Many countries developed therefore programs targeting suicide in adolescents and young adults^12^,^19^,^44^,^45^,^46^,^47^,^48^,^49^,^50^,^51^,^52^,^53^,^54^. Several countries have developed school-based suicide prevention programs^13^,^14^,^55^,^56^,^57^. As suicide attempts were found to be higher in youths homo- and bisexuals, and a specific program was also developed to prevent homophobic bullying in this population in France^58^. New connected technologies have been suggested to improve suicide prevention in this population and need to be further evaluated^59^,^60^,^61^,^62^. The elderly was also described as at risk for suicide^63^. Most elderly suicide prevention programs were centered on the reduction of risk factors (depression screening and treatment, and decreasing isolation), but when gender was considered, programs were mostly efficient for women^18^. Overall, our results suggest that suicide prevention policies were more effective in adolescents/young adults than in the elderly. Only three countries had increased rate of suicide deaths in youths versus nine in the elderly. It may be underlined that four countries with the highest rates of suicide increase in the elderly had low quality data (Armenia +166.7%, Poland +60.6%, Portugal +81%, Greece +10.9%).

One of the major findings of the present work was that suicide mortality in males aged 35–54 years increased, or at least stagnated between 1990 and 2010 (Fig. 3). Several explanations were recently suggested in the literature to explain this phenomenon. Unemployment rate was recently found to be associated with increased suicide mortality between 2000 and 2010, in several European countries^64^. The impact of unemployment on suicide rates was suggested to be higher in males compared to females^61^,^64^,^65^,^66^. It may therefore be suggested that further suicide prevention policies focus on unemployed people. It was also suggested that the recent worldwide financial and economical 2008 crisis may have been associated with increased completed suicides in Western European countries^61^,^67^,^68^,^69^,^70^,^71^. However, the results for the USA suggested that the suicide mortality regularly increased between 1990 and 2010, and no significant suicide mortality increase was noted in European countries. The 2008 crisis can therefore not be argued as the unique factor implicated in suicide mortality increase in this population. The suicide mortality in middle-aged males may also be due to suicide in jail. However the studies on the jail suicide prevalence remain few in number, and the studies on suicide prevention in jail are even more rare. A study assessing suicide rate in the prisons of 12 countries concluded that rates of prison suicide did not reflect general population suicide rates^72^. This suggested that variations in prison suicide rates reflected differences in criminal justice systems including, possibly, the provision of psychiatric care in prison. A national study in the USA concluded that there has been a significant decrease in the rate of suicide in detention facilities during this period^17^. To deal with this public health issue, a peer-prevention program in prisons was recently developed in France^73^. Low socioeconomic status, unhealthy lifestyle habits, difficulties in health care access and poor longitudinal continuity of care were also suggested as potent etiological factors of suicide mortality that could also be targeted by suicide prevention policies^74^,^75^. Finally, another explanation for the increase of suicide mortality in the 35–54 years may be due to the improvement of medical care system. Although most of the suicide deaths were associated with the first attempt^76^, it may be hypothesized that a better management of suicide attempts in youth may be associated with an increase of the mean age at death by suicide. Further studies should assess the impact of suicide interventions on suicide trajectories, beyond the absolute number of deaths by suicide.

When examining the suicide trajectories, we found that Armenia and Greece (group 1) reported lower stable rates of suicide compared to other countries. As quality data was evaluated as “low” for these countries, it may be hypothesized that suicide may be under-reported in these countries. In group 2, which includes countries with stable suicide rates below 10 per 100,000 habitants, only United Kingdom reported high quality data. Most of the other countries were included in group 3, with stable suicide rates between 10 and 15 per 100,000 between 1990 and 2010. Group 4 and 5 included countries with high suicide rates in 1990, with strong decrease between 1990 and 2010. Except for Finland, all these countries were Easter European countries. As suicide rates regularly decreased since 1994, it may be suggested that the collapse of Soviet Union was associated with a decrease of suicide rates in Easter European countries^21^. A high suicide rate (99.8%) in forensic autopsy registers in Finland had been already described^77^. Economic hardship was described as strongly associated with suicide mortality in Finland^78^. It was also recently suggested that Finland ASDRs may be explained by gender differences: In Finland, jumping in front of moving objects and firearms were frequently used by male adolescents^79^. The suicide mortality of Finnish women is currently the second highest in Europe^80^. Antidepressant consumption was not associated with increased suicide attempts in a large Finish 1966 birth cohort^81^. Some seasonal/weather parameters were also suggested to impact suicide death rates in Finland^82^,^83^,^84^ and East European countries^85^.

## Strengths and limitations of study

The cause of death reported in national statistics may be of limited reliability, and WHO quality indices displayed in the table indicate that, for all causes of death combined, data from Armenia, Greece, Poland, and Portugal should be considered with caution. Suicide may often be misclassified as an accident or another cause of death. Registering a suicide is a complicated procedure involving several different authorities, often including law enforcement. Suicide mortality may have been underestimated in these countries. However trained clinicians wrote the death certificates, which may have limited the number of incorrect or incomplete certificates. These data remains mainly descriptive allowing to formulate hypotheses, but without causal certainty. In summary, we are confident that our results do reflect real trends. However, despite this data, it is still difficult to assess the efficacy of suicide programs.

## Conclusion

The evaluation of the effectiveness of suicide prevention policies remains an important goal and challenge for the authorities. Overall, suicide mortality decreased between 1990 and 2010 in Europa and in the USA, and the gender difference was less marked in 2010 compared to 1990. The evolution of suicide in Europa and USA suggested that suicide prevention policies were effective in youths, but less in the elderly. In contrast, suicide mortality increased in the 35–54 years old in half of the studied countries. Key elements in developing a national suicide prevention strategy are to make prevention a multisectoral priority that involves the health sector but also education, employment, social welfare, the judiciary and others. Communities play a critical role in suicide prevention. They can provide social support to vulnerable individuals and engage in follow-up care, fight stigma and support those bereaved by suicide. Health-care services need to incorporate suicide prevention as a core component. Early identification and effective management are keys to ensuring that people receive the care they need. Suicide prevention interventions should be multimodal, evidence based, guided by specific testable hypotheses, and implemented among populations of sufficient size to yield generalizable and reliable results. It would be necessary to develop international assessment programs and the present work can help to get this direction.

## Additional Information

**How to cite this article**: Fond, G. *et al.* Disparities in suicide mortality trends between United States of America and 25 European countries: retrospective analysis of WHO mortality database. *Sci. Rep.* **6**, 20256; doi: 10.1038/srep20256 (2016).

## Figures and Tables

**Table 1:** Changes in suicide mortality between 1990 and 2010 in European countries and United States of America (USA) ranked according to overall decline in mortality.

Countries	ASDR per 100 000 -all ages—both sexes		All ages, both sexes		Females	Males	15–24	25–34	35–54	55–74	75+	Quality of data on cause of death
	1990	2010	Annual	Overall	Overall rate							
Estonia	2.0	13.5	−2.3	−46.0	−39.8	−64.3	−40.5	−56.6	−57.1	−37.0	−26.8	High
Hungary	33.5	18.7	−2.2	−44.2	−40.0	−55.6	−54.0	−51.2	−41.6	−24.8	−47.5	High
Finland	27.5	15.9	−2.1	−42.2	−46.1	−30.3	−33.8	−47.7	−47.6	−10.9	−22.7	High
Austria	19.5	11.4	−2.1	−41.5	−39.1	−51.5	−42.0	−58.0	−24.8	−23.5	−23.9	Medium
Slovenia	24.4	15.1	−1.9	−38.1	−36.3	−51.5	−27.8	−50.7	−33.0	−11.8	0.0	High
Croatia	19.8	12.7	−1.8	−35.9	−33.5	−45.1	−19.7	−60.5	−32.6	−32.7	−7.1	Medium
Bulgaria	12.3	7.9	−1.8	−35.8	−28.4	−49.3	−62.9	−50.0	−21.0	−36.0	−27.6	Medium
Germany	13.7	8.8	−1.8	−35.8	−33.7	−44.6	−41.1	−52.5	−23.0	−16.1	−30.7	Medium
Czech Republic	16.7	11.4	−1.6	−31.7	−26.3	−55.4	−14.3	−15.3	−22.3	−17.7	−52.6	Medium
Latvia	23.4	16.0	−1.6	−31.6	−26.7	−57.8	−24.0	−53.1	−34.6	−21.6	−54.5	High
Sweden	14.3	10.1	−1.5	−29.4	−27.1	−37.9	0.0	−37.7	−23.4	−13.4	−34.8	Medium
Norway	14.0	10.1	−1.4	−27.9	−32.7	−15.7	−23.4	−22.5	−9.5	−17.8	−16.3	Medium
Armenia	3.0	2.3	−1.2	−23.3	−18.6	−33.3	−23.1	−45.0	−50.0	17.6	166.7	Low
France	16.8	13.1	−1.1	−22.0	−21.3	−27.0	−36.5	−42.4	10.4	−4.8	−13.5	Medium
Italy	6.0	4.8	−1.0	−20.0	−15.1	−35.5	−52.1	−26.4	24.2	−13.5	−15.9	Medium
Spain	6.2	5.0	−1.0	−19.4	−17.3	−29.0	−58.5	−20.2	92.0	−13.6	5.0	Medium
United Kingdom	7.3	6.0	−0.9	−17.8	−18.3	−15.6	−26.6	−31.5	14.8	−12.5	−24.4	High
Greece	2.9	2.6	−0.5	−10.3	−2.1	−53.8	−60.4	3.5	52.7	3.2	10.9	Low
Netherlands	8.4	8.0	−0.2	−4.8	3.6	−23.3	−11.5	−42.7	39.1	43.9	−26.9	Medium
Portugal	7.4	7.1	−0.2	−4.1	−3.4	−5.7	−55.2	−36.1	60.2	25.8	81.1	Low
USA	11.4	11.0	−0.2	−3.5	−7.8	2.2	−5.5	−12.4	62.9	41.5	−4.6	High
Belgium	15.7	15.3	−0.1	−2.5	−−1.7	−11.8	−11.0	−20.4	42.3	−2.4	−18.8	Medium
Ireland	10.0	10.4	0.2	4.0	12.0	−16.0	39.3	32.4	78.9	16.7	18.2	High
Poland	12.7	13.7	0.4	7.9	13.8	−21.4	16.4	−15.3	29.5	65.7	60.6	Low
Lithuania	24.3	27.6	0.7	13.6	14.8	2.4	72.6	−29.2	2.4	8.0	45.2	High
Romania	8.4	10.6	1.3	26.2	43.0	−21.4	−12.6	12.0	47.5	44.7	32.3	High
Summary statistics												
Median	13.85	10.80	−1.1	−22.7	−20.0	−31.8	−25.3	−36.9	−3.6	−12.2	−16.1	
Min	2.90	2.30	−2.3	−46.0	−46.1	−64.3	−62.9	−60.5	−57.1	−37.0	−54.5	
Max	33.50	27.60	1.3	26.2	43.0	2.4	72.6	32.4	92.0	65.7	166.7	

ASDR Age−standardized Death Rate.
